# The Current Landscape of Hypotheses Describing the Contribution of CD4+ Heterogeneous Populations to ALS

**DOI:** 10.3390/cimb46080465

**Published:** 2024-07-23

**Authors:** Mariusz Sacharczuk, Michel-Edwar Mickael, Norwin Kubick, Agnieszka Kamińska, Jarosław Olav Horbańczuk, Atanas G. Atanasov, Piotr Religa, Michał Ławiński

**Affiliations:** 1 Institute of Genetics and Animal Biotechnology, Polish Academy of Sciences, Postępu 36A, 05-552 Jastrzębiec, Poland; m.sacharczuk@igbzpan.pl (M.S.); j.horbanczuk@igbzpan.pl (J.O.H.); atanas.atanasov@dhps.lbg.ac.at (A.G.A.); m.lawinski@igbzpan.pl (M.Ł.); 2 Department of Pharmacodynamics, Faculty of Pharmacy, Medical University of Warsaw, Banacha 1B, 02-091 Warsaw, Poland; 3 Department of Biology, Institute of Plant Science and Microbiology, University of Hamburg, Ohnhorststr. 18, 22609 Hamburg, Germany; kubick.norwin@googlemail.com; 4 Faculty of Medicine, Collegium Medicum Cardinal Stefan Wyszyński University in Warsaw, 01-938 Warsaw, Poland; agnieszka.kaminska@uksw.edu.pl; 5 Ludwig Boltzmann Institute Digital Health and Patient Safety, Medical University of Vienna, 1090 Vienna, Austria; 6 Department of Laboratory Medicine, Division of Pathology, Karolinska Institute, SE-141 86 Stockholm, Sweden; piotr.religa@ki.se; 7 Department of General Surgery, Gastroenterology and Oncology, Medical University of Warsaw, 02-091 Warsaw, Poland

**Keywords:** CD4, Amyotrophic Lateral Sclerosis, Th17, Treg

## Abstract

Amyotrophic Lateral Sclerosis (ALS) is a poorly understood and fatal disease. It has a low prevalence and a 2–4 year survival period. Various theories and hypotheses relating to its development process have been proposed, albeit with no breakthrough in its treatment. Recently, the role of the adaptive immune system in ALS, particularly CD4+ T cells, has begun to be investigated. CD4+ T cells are a heterogeneous group of immune cells. They include highly pro-inflammatory types such as Th1 and Th17, as well as highly anti-inflammatory cells such as Tregs. However, the landscape of the role of CD4+ T cells in ALS is still not clearly understood. This review covers current hypotheses that elucidate how various CD4+ T cells can contribute to ALS development. These hypotheses include the SWITCH model, which suggests that, in the early stages of the disease, Tregs are highly capable of regulating the immune response. However, in the later stages of the disease, it seems that pro-inflammatory cells such as Th1 and Th17 are capable of overwhelming Treg function. The reason why this occurs is not known. Several research groups have proposed that CD4+ T cells as a whole might experience aging. Others have proposed that gamma delta T cells might directly target Tregs. Additionally, other research groups have argued that less well-known CD4+ T cells, such as Emoes+ CD4+ T cells, may be directly responsible for neuron death by producing granzyme B. We propose that the ALS landscape is highly complicated and that there is more than one feasible hypothesis. However, it is critical to take into consideration the differences in the ability of different populations of CD4+ T cells to infiltrate the blood–brain barrier, taking into account the brain region and the time of infiltration. Shedding more light on these still obscure factors can help to create a personalized therapy capable of regaining the balance of power in the battle between the anti-inflammatory and pro-inflammatory cells in the central nervous system of ALS patients.

## 1. Introduction 

Amyotrophic Lateral Sclerosis (ALS) is a fatal disease that affects the nerve cells in the brain and spinal cord mortality [1,2,3]. Roughly one-third of ALS patients experience bulbar symptoms such as dysphagia or dysarthria, while a smaller percentage have respiratory issues. Moreover, nearly 50% of ALS patients have some form of cognitive impairment, and up to 15% of patients meet the criteria for frontotemporal dementia [4,5]. The global prevalence of this disease is around 5 per 100 thousand people, with an expected period of survival of 4 years. Further stratification of patient data shows that 34.1% of ALS patients die within 12 months, 50% die within 30 months of the onset of symptoms, and about 20% of patients survive between 5 to 10 years after the onset of symptoms [6,7]. ALS includes two main types: sporadic and familial [8,9]. Sporadic ALS accounts for the majority of cases and occurs randomly with no known cause, while familial ALS is inherited and runs in families [10,11]. The peak age at onset is reported to be between 58 and 63 years for sporadic disease and 47 to 52 years for familial disease [6]. The genes controlling familial ALS are diverse, with mutations in multiple genes associated with the disease. However, mutations in four genes (C9orf72, SOD1, TARDBP, and FUS) account for 70% of the cases [12]. The general inheritance pattern is in an autosomal dominant manner, where inheritance of one copy of the ALS-associated mutated genes can result in a 50% probability of inheriting the condition. However, both types share the same symptoms [13,14,15].

Currently, there is no cure for ALS [7]. Riluzole is the most widely used treatment. It can increase the probability of one-year survival by 9%, highlighting the limited treatment options available to ALS patients [12,16,17]. Although the riluzole mechanism of action is not fully understood, studies have shown that it inhibits glutamate-induced neuron damage by inhibiting its release [18,19]. Over 60 potential ALS treatments that offer anti-inflammatory, antioxidant, and neuroprotective bioactivities have been investigated, with most failing to demonstrate efficacy in clinical trials. In 2017, Edaravone was approved for treating ALS in Japan, South Korea, and the US. Edaravone is an antioxidative compound that reduces oxidative stress by scavenging oxygen radicals within the central nervous system, with patients showing reduced functional loss after 6 months [20,21]. Alternative approaches, including RNA-based therapeutics targeting the most common ALS genes—SOD1, C9ORF72, FUS, and ATXN2—using small activating RNAs (saRNA) [22]. The saRNA approach aims to modulate gene expression on the non-mutated allele [22]. These saRNAs can induce gene expression at a transcriptional level by targeting promoter sequences or gene antisense transcripts [22]. Currently, there are no saRNA treatments approved for ALS clinical trials. Other therapies include viral vectors such as adeno-associated virus are gaining momentum [23,24,25]. However, these strategies could prove risky for ALS patients [22,26].

Studies show that CD4+ T cells play a fundamental role in ALS. Although several theories have been formulated, the pathophysiology of ALS remains unclear. Such theories are linked with mitochondrial dysfunction, superoxide dismutase gene mutations, and abnormalities in neuronal glutamate transports, and some reports even postulated about the role of viruses such as HIV [27,28,29]. Recently, several investigations have revealed evidence of activated microglia, IgG deposits, and dysregulated cytokine expression in the spinal cords of ALS patients, raising the possibility that the immune system, and in particular CD4+ T cells, may play a proactive role in the disease process [30]. For example, studies have observed increased peripheral CD4+ (and CD8+) T cells in ALS patients compared to healthy controls. CD4+ T cells in ALS patients also exhibit increased activation, which may coincide with impaired intrathecal regulation by CD56(bright) NK cells, potentially contributing to increased disease progression [31]. Additionally mice that lacked CD4+ T cells but had the mSOD1^G93A^ mutation (i.e., mSOD1^G93A^/RAG2^−/−^) showed an acceleration of the disease progression [32]. This review explores the current body of evidence detailing the roles of CD4+ T cells in ALS and the hypotheses driving the search for ALS treatment options.

## 2. Current Understanding of CD4+ T Cells Populations

CD4+ T cells are a phenotypically diverse group of cells with different functions [33]. They are produced in the bone marrow, followed by maturation in the thymus [34,35,36,37]. Naive CD4+ T cells migrate to the periphery, where they differentiate. T cells can be phenotyped based on the type of cytokines they produce. Interferon-gamma (IFN-γ) and interleukin 2 (IL-2) are mostly secreted by type 1 T-helper cells (Th1 cells), whose function is mediated by T-box transcription factor (Tbet) [38]. Th2 cells that primarily produce IL-4, IL-5, and IL-13 are regulated by GATA Binding Protein 3 (GATA3) [39]. Organ-specific autoimmune disorders are linked to Th1 cells, whereas Th2 cells are linked to allergies. In the traditional model, Th1 cells target cancer cells and can trigger a delayed-type hypersensitivity (DTH) skin response against bacterial and viral antigens, focusing on fighting intracellular pathogens such as viruses [40]. Th2 cells, on the other hand, prioritize defense against external pathogens, including multicellular parasites [41]. The Th1 pathway tends to produce a pro-inflammatory response and thus is frequently described as being the more aggressive of the two. Th2 cells appear to possess several anti-inflammatory capabilities, suggesting that Th1 and Th2 regulate each other [42,43].

Similarly, T-helper 17 (Th17) cells and regulatory T cells (Tregs) are modeled as forming the Th17/Treg axis. Th17/Treg axis formulation is based on the observation that, while Th17 and Treg have a similar transcriptome, their functions are different [44,45]. The transcription factor RAR-related orphan receptor-γ (RORγt) is the main regulator of Th17 cells [46,47]. Th17 cells are critical for the development of pathological autoimmunity [46]. Th17 cells generate various pro-inflammatory cytokines, such as IL-1, IL-6, IL-17A, TNFα, and IFNγ. They have also been shown to play a key role in the pathophysiology of various neurodegenerative diseases such as Parkinson’s and Alzheimer’s, as well as mental disorders like depression [48]. Autoreactive T cells are thought to be suppressed by FoxP3+ Treg cells, which are regulatory CD4+ T cells [49]. Treg cells can directly and indirectly suppress other CD4+ T cells. Direct regulatory strategies involve the synthesis of ICER, CD39, IL-4, and IL-10 [49]. One example of indirect techniques includes the use of the expression of IL-2R (CD25) to consume IL-2, which is vital for other CD4+ T cells’ development [49]. 

There are various less-studied populations of CD4+ T cells. These include Th9 and Th22 and several intermediate CD4+ T cells, such as Tr1 and RORγt+ FoxP3+ Tregs. Eomesodermin (EOMES)+ CD4 T cells are another less characterized type of CD4 T cells [50,51,52]. These less well-known populations have not been fully studied in the context of ALS. Further investigations to dissect their contribution could yield valuable insights into disease development.

Several studies have shown that CD4+ T cells can infiltrate the focally damaged BBB [44,46]. One piece of evidence supporting the critical role of CD4+ T cells in ALS is the status of the BBB, wherein ALS progression leads to significant focal damage, as defined by the Zlokovic–Cleveland Model [53,54,55]. This model postulates that during ALS, edema and serum protein leakage (e.g., albumin and immunoglobulins (Igs)) result in focal tissue hypoxia. Red blood cell (RBC) extravasation releases neurotoxic haemoglobin (Hb)-derived products focally in the spinal cord tissue [56]. Free Hb is directly toxic to motor neurons through the generation of reactive oxygen species (ROS). It is hypothesized that focal Ig leakage promotes the activation of microglia and astrocytes, contributing to cell death. The leakage of Ig, which interacts with motor neuron antigens, also exerts direct toxic effects on motor neurons [57]. The ability of CD4+ T cells to cross the BBB was shown in a rodent SOD1ALS mouse model where their infiltration into the midbrain–interbrain region was confirmed via a live magnetic resonance approach [58]. The T cell influx contributes to the further activation of astrocytes; this is a hypothesis supported by animal and human studies. For example, transgenic mSOD1G93A mice mated with immunodeficient mice, and bone marrow transplantation (BMT) was utilized in selective reconstitution to clarify the functions of T cells. These results showed that CD4 T lymphocytes activated microglia and astrocytes. They also promoted neuroprotection by altering the glial balance between trophism and cytotoxicity. However, the authors did not examine the difference in the effect of various CD4+ T cells [32]. 

## 3. Hypotheses Exploring the Role of CD4 in ALS

Building upon the information that CD4+ T cells are capable of infiltrating the BBB during ALS, current research is focused on four feasible hypotheses that aim to form an integrative model of how CD4+ T cells are involved in ALS. 

### 3.1. The SWITCH Hypothesis 

Recent research demonstrated that CD4+ T cell subtypes contribute differently to ALS development. In their report, Ford et al. compared the levels of Th1-associated cytokines, such as interleukin-12 (IL-12) and interferon-gamma (IFNγ), present in the cerebrospinal fluid (CSF) of ALS patients with those of patients suffering from other neurological diseases. All samples were below the detection limit for IL-12 and IFNγ [59]. Holmoy et al. challenged these findings by extracting and cloning CD4+ T cells from ALS patients [60]. The cloned CD4+ T cells predominantly exhibited Th1-like behavior, producing IFNγ. Th1 cells are known to induce a microglia pro-inflammatory phenotype which can contribute to the inflammation status of the CNS in ALS patients, generating pro-inflammatory molecules such as tumor necrosis factor-alpha (TNFα); interleukin IL-6, IL-1β, and IL-12; and chemokine ligand-2 (CCL2) [61,62]. Microglia can also support the activation of Th1 by playing the role of antigen-presenting cells [63]. It is worth noting that astrocytes seem to increase Th1 differentiation [64]. However, the direct effect of Th1 on astrocytes in the context of ALS is not yet clear.

Shi et al. investigated the presence of CD4+ T cells in 21 ALS patients, 14 spinocerebellar degenerative disease control (DC) patients, and 16 healthy controls (HC) [65]. The only T cell subset that showed significantly higher levels in ALS patients than in DC and HC patients was the group known as CD4+ IL-13+ T cells. This subset is typically associated with Th2 cells. CD4+ IL-13+ T cell frequency was negatively associated with the modified ALS functional rating scale score while positively correlated with disease progression, suggesting that IL-13 may play a role in ALS. These differences could result from variations in the frequency of Th1 and Th2 cells among brain regions. Beers et al. showed that, in SOD ALS mice, IL-4 levels were higher in the cervical than in the lumbar spinal cords. The mRNA level of the transcription factor Gata-3 (regulating Th2 response) was elevated in the cervical cord of ALS mice, whereas T-Bet (regulating Th1 response) was increased in the lumbar cord [66]. However, the effect of Th2 on the CNS through the production of IL-4 is debatable as the impact of IL-4 on astrocytes can vary, displaying either pro-inflammatory or anti-inflammatory properties based on the different treatment methods and timing conditions emplyoed [67].

Th17 cells play a crucial role in ALS development through the production of IL-17 [68,69]. Interleukin-17, the main cytokine produced by Th17 cells, is upregulated in the serum of both familial and sporadic ALS patients [70,71]. IL-17 and IL-23 are also upregulated in the CSF of ALS patients [70]. One ALS mutation that leads to C9ORF72 deficiency is linked to increased IL-17A [72]. Compared to Alzheimer’s disease patients, sporadic ALS (sALS) patients have significantly higher levels of IL-6 and sIL-6R in their cerebrospinal fluid, which is needed for Th17 activation [73]. Further evidence supporting the role of Th17 cells includes the finding that targeting CD40L, a crucial co-stimulator for Th17 in SOD1 knockout mice, slows weight loss and paralysis and extends the survival period [74]. A key characteristic of ALS is a high degree of astrogliosis [75,76,77]. We found that Th17 infiltration into the brain is associated with astrogliosis [46]. Once inside the brain, Th17 cells may induce astrogliosis through a cycle of the over-activation of astrocytes, producing IL-6 and IL-1 [78,79]. 

Treg cells seem to play an anti-inflammatory role in ALS. The reconstitution of mSOD1 mice using ex vivo Treg cells from donor WT mice increased survival and postponed the loss of motor function [80,81]. Luchi et al. examined the difference in the frequency levels of Tregs between SOD1+/+ and SOD1−/− mice at five weeks of age and could not identify any significant difference [82]. Treg numbers increase at the early stages of the disease [3]. They also produce IL-4 and are associated with M2-mediated neuroprotection [83,84]. Rentzos et al. compared the number of Tregs in the blood samples of control subjects and ALS patients. Their research showed that Treg numbers in ALS patients are significantly lower than their control counterparts. They also observed that Treg cell numbers are negatively correlated with the progression of the disease [85]. Increased levels of CD4+FOXP3− effector T cells in the blood and CSF are linked to decreased survival, but increased levels of activated Treg cells and the ratio of activated to resting Treg cells in the blood are linked to increased survival [86]. These results are consistent with the findings of Mantovani et al., who observed increased levels of CD4+ cells in the blood of ALS patients compared to healthy controls. They also found that Treg levels were reduced in patients at a less severe stage of the disease [87]. Regulatory T cells (Tregs) can regulate astrocyte reactivity by suppressing the presence of C3-positive astrocytes associated with inflammation in favor of promoting A2-like phenotypes, known for their neuroprotective properties [88]. One hypothesis is that Treg cells play a protective role by producing TGFβ and IL-10 and suppressing Th1 and Th17 CD4+ T cells in ALS [3,89].

**Figure 1:**
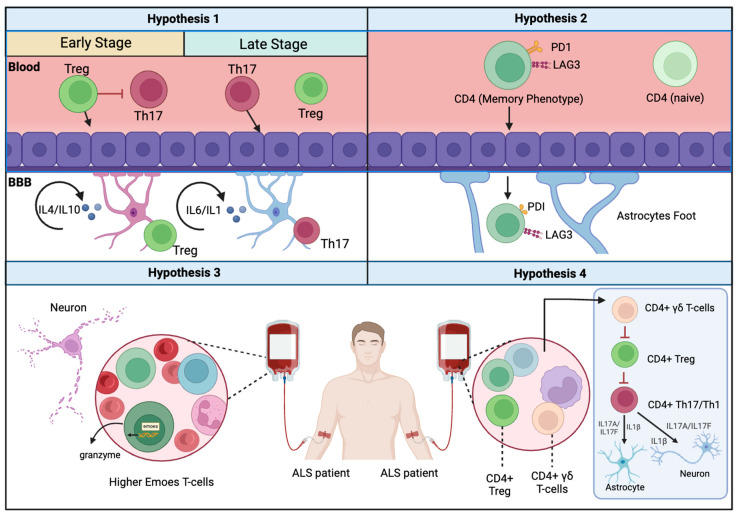
Summary of four plausible hypotheses for the description of CD4+ T cells in ALS. The SWITCH hypothesis assumes that Treg loses its capacity to suppress Th17 in the later stages of the disease. Hypothesis 2 underscores the effect of the aging of memory CD4+ T cells on the progression of ALS. Hypothesis 3 focuses on the role of specific, less-known CD4+ T cells that can harm neurons, while hypothesis 4 underlines the effect of γδ CD4+ T cells in terms of inhibiting the anti-inflammatory effects of CD4+ Tregs.

One hypothesis that aims to put together the pieces of the puzzle is the “SWITCH” model. The critical aspect of this model is the function of time, where it is assumed that Treg can exert a sufficient protective role in ALS, but only at the earlier stages of the disease. However, as the disease progresses, Treg cells can be overwhelmed by the massive infiltration of pro-inflammatory CD4+ T cells into the CNS (Figure 1). Henkel et al. demonstrated that FoxP3 protein expressions were decreased and had an inverse correlation with the rates of advancement of the disease [83]. These findings were also validated for other anti-inflammatory mRNAs, such as TGF-β, and IL-4 [90,91,92]. These findings were also validated for other anti-inflammatory mRNAs, such as TGF-β and IL-4. The SWITCH model was further supported by analyzing chemokine profiles that showed a switch between the neuroprotective response associated with Treg to a more neurotoxic immune response. For example, pro-inflammatory chemokine/cytokine expression was increased in ALS sera, while anti-inflammatory chemokines/cytokines remained unchanged or decreased [93]. Recently, a study examined the immune profiles of 73 ALS patients and 48 healthy controls in peripheral blood and cerebrospinal fluid samples [94]. The results showed that, as the disease progressed, a shift towards pro-inflammatory Th1 and Th17 cells was seen in peripheral blood, while the levels of anti-inflammatory Th2 and T regulatory cells decreased. Pro-inflammatory serum cytokines increased, while the presence of anti-inflammatory cytokine IL-10 decreased. Correlation analysis showed moderate negative correlations between Th1 and Th17 with the ALS functional rating scale revised and forced vital capacity.

### 3.2. The CD4+ T Cell Aging Hypothesis 

Multiple studies have shown that CD4+ T cells are significantly affected by natural aging. A key characteristic associated with aging is thymus involution, in which both the size and function of the thymus are reduced [95,96]. These changes cause a shift in the ratio between naïve and memory CD4+ T cells. This shift can be attributed to a reduction in the thymic output of de novo naïve immune cells and the fact that some existing naïve immune cells acquire a memory cell phenotype [97]. Additionally, T cell receptor excision circle (TREC) molecules—extrachromosomal circular DNA fragments formed as a byproduct of the rearrangement process that creates the T cell receptor (TCR)—are also significantly reduced [98,99]. While the number of regulatory T cells (Tregs) has been reported to increase with age, the overall inducibility of Tregs decreases along with the general functionality of the immune system, resulting in chronic inflammation and a higher risk of autoimmune diseases [100,101,102,103]. 

In the context of late-stage Amyotrophic Lateral Sclerosis (ALS), there is growing evidence that CD4+ T cells infiltrating the blood–brain barrier exhibit signs of advanced aging (Figure 1). Shir Zaccai et al.’s study of the CD4+ T cell profile in the SOD1G93A mouse model of late-onset ALS showed that a high proportion of activated CD4+ T cells expressed PD-1 and LAG-3, known markers of aging in CD4+ T cells [104]. In humans, the level of PD-1 was found to be significantly higher in ALS patients compared to the controls, suggesting that aging in CD4+ T cells might contribute to their behavior in ALS cases [105]. However, several questions remain unanswered. First, what drives the aging process in CD4+ T cells? Second, why does the aging process of CD4+ T cells progress rapidly in ALS? Moreover, how does this model align with the SWITCH model, which suggests a decrease in the number of Tregs in later stages of ALS. Future research exploring these aspects may provide a better understanding of the complex role of CD4+ T cells in ALS [104].

### 3.3. The Network of Various CD4+ T Cells Interaction Hypothesis 

The development of ALS involves the role of less-understood CD4+ T cells, such as Eomes+ T helper cells. These T helper cells are cytotoxic and express the T-box transcription factor Eomesodermin (Eomes). Deleting the Eomes gene in T cells has improved the prognosis of late-onset experimental autoimmune encephalomyelitis (EAE). This unique group of cells is abundant in the cerebrospinal fluid (CSF) of multiple sclerosis patients [106,107]. Studies have demonstrated that Eomes+ T helper cells can release granzyme B. Once granzyme B has been internalized by other cells, its presence can induce apoptosis by activating caspases (e.g., caspase-3) [108,109]. The flow cytometry analysis of immune cells from the brain tissue of postmortem progressive multiple sclerosis individuals revealed a high frequency of Eomes+ CD4+ T cells [108]. The recent phenotyping of 28 peripheral blood samples from ALS patients found that the frequency of Eomes+ T helper cells was significantly increased in ALS compared to age-matched healthy controls, especially during the initial phase of the disease [110]. Additionally, granzyme B production by these T helper cells was higher in the patient group when compared to the controls. These intriguing findings open the door for further research into the relationship between various CD4+ T cells, the timing of their entry into the central nervous system (CNS), and disease progression. Moreover, the molecular pathways employed by Eomes+ T helper cells within the CNS microenvironment—including interactions with neural cells and other CD4+ T cells—remain to be elucidated.

### 3.4. The γδ T Cell Hypothesis

Among the other lines of research that investigators have been exploring is the role of the γδ T cells in ALS [111]. This peculiar group of cells is mostly CD4^−^CD8^−^, and their γδ T cell TCR complex includes two protein chains, γ cell, and δ instead of the traditional α and β that appear on the surface of other T cells [112,113,114]. γδT cells can recognize a wide variety of antigens, and they do not require antigen presentation by MHC I or MHC II as they can recognize antigens in a non-MHC-restricted manner [115]. One of the main molecules substituting for the function of antigen presentation by MHC to the gamma delta cells is CD1 [116]. There are five CD1 isoforms in humans (CD1a, CD1b, CD1c, CD1d, and CD1e), each with a different distribution and antigen presentation profile [117,118]. Among the different CD1 isoforms, CD1d has been shown to present glycolipid antigens to γδ T cells [118]. γδ T lymphocytes can kill Tregs through CD1d [119]. CD1 is expressed by immune cells in the brain. A recent study by Xiaoyan Li et al. examined γδ T cell association with ALS [111]. The authors used mass cytometry to analyze the immune profile of cells isolated from ALS patients [111]. They found that γδT cells were associated with the fast-progressing form of ALS. However, these findings raise more questions; for example, the exact function of the γδ T cells in ALS is still unknown, and their interaction with the CD1 molecular complex within the ALS context has not yet been proven. We hypothesize that Treg loss in ALS could be related to the targeting of Tregs by γδ T cells. Furthermore, uncovering the antigens that activate the function of this group of cells can help to identify the link between ALS and autoimmune disorders. 

## 4. Current Therapy Targeting CD4+ T Cells in ALS 

Currently, various clinical trials are exploring hypotheses (Table 1) [120]. Thonhoff et al. demonstrated that an autologous Treg infusion in ALS patients effectively slowed down the disease progression [121]. However, the study’s sample size was limited to three patients, which affected its generalizability [122]. Subsequently, another trial with eight patients treated with Treg and IL-2 showed similar results [123,124]. A phase II trial using low-dose IL-2 achieved comparable outcomes [125]. Ongoing trial study topics that include the use of fecal microbiota transplants to enhance Treg production [126]. A potential research avenue yet to be explored would be targeting Th17 migration in the initial disease phase and subsequently enhancing Treg function in the later phase. Henderson et al. investigated the safety of anti-CD19, but did not specifically assess its impact on CD4+ or its phenotype. Rapamycin has been explored as a clinical treatment for ALS, with reported effects on Th1 frequency [127]. However, its specific impact on each CD4+ T cell subpopulation remains undocumented.

Another method of targeting Tregs is chimeric antigen receptor (CAR) technology [128]. CAR T cell technology involves constructing chimeric proteins composed of three main parts: a single-chain variable fragment derived from an antibody, a transmembrane domain, and an intracellular domain [129,130]. The single-chain variable fragment targets and binds to specific extracellular antigens, while the transmembrane domain stabilizes the structure of the complex [131,132]. The intracellular domain, such as CD3ζ, activates the T cell once the chimeric complex recognizes unique antigens [133,134]. One advantage of this technology is that it does not rely on MHC activation, allowing it to target various antigens [135]. Graber et al. constructed a unique CAR (DG05-28-3z) comprising a single-chain variable fragment that targets human SOD1, a transmembrane domain, a co-stimulatory domain, and a CD3ζ signaling domain [128]. The DG05-28-3z-expressing Tregs produced IL-10 when cultured with aggregated hSOD1G93A proteins or spinal cord explants from hSOD1G93A transgenic mice, but not when cultured with lung or liver tissue explants from the same mice. Co-culturing DG05-28-3z CAR Tregs with human monocytes/macrophages inhibited the production of TNF-α and ROS. However, neither in vivo nor human trials have been reported that use this approach. One possible modification to this method could involve designing CAR Tregs that specifically target the Th17 and Th1 cells migrating to the brain during ALS. This approach could reduce the expression of pro-inflammatory cytokines caused by pro-inflammatory CD4+ T cell migration.

## 5. Challenges to Current Hypotheses

The hypotheses presented above seem plausible. However, several aspects need to be investigated further. First, the temporal order of the CD4+ T cell infiltration of the BBB role can be disputed. Recent research challenges these findings. Andrés-Benito et al. investigated altered the expression of inflammation-associated transcripts in the peripheral blood of early-stage ALS patients [136]. They analyzed the RNA expression levels of 45 genes in 22 sALS patients and 13 age-matched controls using RT-qPCR, while concurrently assessing serum and clinical parameters. Surprisingly, sALS cases exhibited the downregulation of certain chemokines (CCL5 and CXCR5), anti-inflammatory cytokines (IL-10, TGFB2, and IL-10RA), pro-inflammatory cytokines (IL-6), and T cell regulators (CD2 and TRBC1). Moreover, genes involved in leukocyte extravasation (ITGB2, INPP5D, SELL, and ICAM1) and extracellular matrix remodeling (MMP9 and TIMP2) were upregulated. Additionally, during the early disease phase, Tregs may encounter difficulty penetrating the blood–brain barrier (BBB), known for its limited permeability compared to Th17 cells [44]. Furthermore, Beers et al. highlighted Treg dysfunction in both slowly and rapidly progressing ALS patients [137]. These findings contradict the SWITCH model of Treg, which shows a higher ability to infiltrate the BBB in the early stages of the disease. 

One solution to this dilemma could be related to the location of the infiltration; the blood–brain barrier is not uniform in permeability, and some regions have a stronger barrier than others. There might be favored areas for penetration of pro-inflammatory and anti-inflammatory CD4+ T cells, based on the resistance imposed on them and their mode of infiltration or dispersion. For example, Th17 cells are known to prefer a paracellular route, while Th1 and possibly Treg cells follow a transcellular route [44,78].

Another solution could lie in the variability of cytokine effects on ALS development. IL-6 appears to play a crucial role in the pathogenesis and progression of ALS [138]. However, Martinez et al. conducted an intriguing study investigating the cytokine networks involved in disease progression [139]. Their findings revealed that, in progressive cases, IL-4 and IL-6 were negatively associated with disease progression, whereas in patients with longer survival times concentrations of these cytokines were positively correlated with disease progression. This information suggests that clustering patients based on disease type can uncover additional mechanisms influenced by the Th17/Treg axis in the context of ALS.

One of the major obstacles to understanding the role of CD4+ T cells in ALS is using the most appropriate animal model. Transgenic SOD1 rodent models have been used to study ALS biology and potential therapeutics, but translation into human clinical trials has been poor [140]. TDP-43 rodent models show distinct disease phenotypes from human ALS patients, but no correlation exists between rodent and human symptoms. FUS rodent models offer insights into FUS-ALS pathology but do not reproduce human neuropathological features [141]. Wobbler mice share phenotypic features with human ALS [142]. Alternative animal models for ALS, such as pigs and primates, are being explored for disease characterization and the development of therapy. Pigs show severe ALS-like phenotypes and the mislocalization of TDP-43, while primates reveal protein interactions not seen in rodent models [143,144]. Other options that could also be used include induced Pluripotent Stem Cells (iPSCs) and olfactory stem cell cultures [145,146]. The extensive investigation of CD4+ T cells using one or more variants of the animal models may yield a better understanding of ALS development and progression.

## 6. Conclusions

ALS development is a function of a large matrix of complex networks of molecular interactions that are largely affected by CD4+ T cell dysfunction. In ALS, the BBB loses its integrity, thus giving CD4+ T cells the opportunity to invade areas of the CNS that were previously privileged. Upon entry, CD4+ T cells are shifted toward dysfunctional phenotypes, leading to direct damage to neurons or indirect damage via the inhibition of the function of anti-inflammatory Tregs. Future therapies might need to address the critical battle between anti- and pro-inflammatory CDD4+ T cells in the CNS of ALS patients in order to achieve greater success.

## Figures and Tables

**Table 1:** Selected Clinical Trials on ALS.

Title	Study	Status	Intervention	Proposed Target
Study in ALS With Abatacept & IL-2	NCT06307301	Phase I	Drug: Abatacept injection [Orencia] and Proleukin (aldesleukin)	CD28 on T cells
Gilenya in Amyotrophic Lateral Sclerosis (ALS)	NCT01786174	Phase I	Drug: Gilenya Other: placebo	Sphingosine 1-phosphate receptor on Lymphocyte migration to the BBB
Immuno-modulation in Amyotrophic Lateral Sclerosis- a Phase II Study of Safety and Activity of Low Dose Interleukin-2	NCT02059759	Phase II	Drug: 1.0 MIU IL-2 per day Drug: 2.0 MIU IL-2 per day Drug: placebo	T cells and T regulatory cells
Regulatory T Cells for Amyotrophic Lateral Sclerosis	NCT05695521	Phase I	Biological: CK0803 Other: excipient	TGFβ1 and TGFβ2; enhances Tregs
Immunosuppression in Amyotrophic Lateral Sclerosis (ALS)	NCT01884571	Phase II	Drug: Basiliximab Drug: Methylprednisolone Drug: Prednisone Drug: Tacrolimus Drug: Mycophenolate mofetil	Functional inhibition of IL-2
Rapamycin Treatment for ALS	NCT03359538	Phase II	Drug: Rapamycin Drug: placebo oral tablet	Disrupts cytokine-induced T cell differentiation
Nebulized RNS60 for the Treatment of Amyotrophic Lateral Sclerosis	NCT02988297	Phase II	Drug: RNS60 Drug: placebo	Mitochondrial biogenesis, neuroprotection, reduction in inflammation, and increase in Tregs
Phase II/III Randomized, Placebo-controlled Trial of Arimoclomol in SOD1 Positive Familial Amyotrophic Lateral Sclerosis	NCT00706147	Phase II/III	Drug: Arimoclomol Drug: placebo	Increase in heat shock protein (HSP-70) levels with anti-apoptotic action
HEALEY ALS Platform Trial - Regimen B Verdiperstat	NCT04436510	Phase II/III	Drug: Verdiperstat Drug: matching placebo	Reduction in oxidative stress and neuroinflammation
Perampanel for Sporadic Amyotrophic Lateral Sclerosis (ALS)	NCT03019419	Phase II	A Drug: Perampanel Drug: placebo	A non-competitive selective antagonist of postsynaptic ionotropic alpha-amino-3-hydroxy-5-methyl-4-isoxazolepropionic acid (AMPA) glutamate receptor
